# Computationally-guided optimization of small-molecule inhibitors of the Aurora A kinase–TPX2 protein–protein interaction†
†Electronic supplementary information (ESI) available: Computational methods and structure files, chemical synthesis, fluorescence polarization assays, crystallographic data. See DOI: 10.1039/c7cc05379g


**DOI:** 10.1039/c7cc05379g

**Published:** 2017-08-02

**Authors:** Daniel J. Cole, Matej Janecek, Jamie E. Stokes, Maxim Rossmann, John C. Faver, Grahame J. McKenzie, Ashok R. Venkitaraman, Marko Hyvönen, David R. Spring, David J. Huggins, William L. Jorgensen

**Affiliations:** a Department of Chemistry , Yale University , New Haven , Connecticut 06520-8107 , USA; b School of Chemistry , Newcastle University , Newcastle upon Tyne NE1 7RU , UK . Email: daniel.cole@ncl.ac.uk; c MRC Cancer Unit , University of Cambridge , Hills Road , Cambridge CB2 0XZ , UK; d Department of Chemistry , University of Cambridge , Lensfield Road , Cambridge CB2 1EW , UK; e Department of Biochemistry , University of Cambridge , 80 Tennis Court Road , Old Addenbrooke's Site , Cambridge CB2 1GA , UK; f Theory of Condensed Matter Group , Cavendish Laboratory , 19 JJ Thomson Avenue , Cambridge CB3 0HE , UK

## Abstract

Free energy perturbation theory, in combination with enhanced sampling of protein–ligand binding modes, is evaluated in the context of fragment-based drug design, and used to design two new small-molecule inhibitors of the Aurora A kinase–TPX2 protein–protein interaction.

## Abstract 2

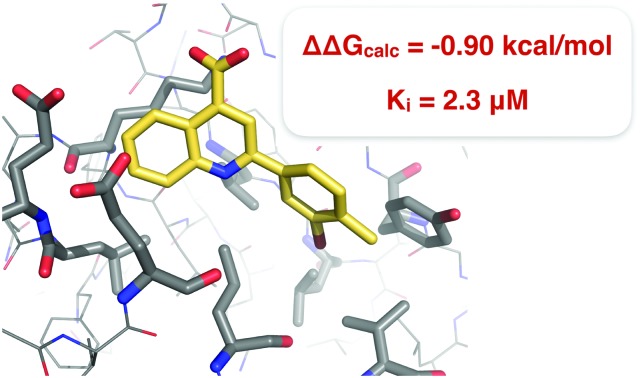
Computational binding free energy predictions were validated against experiment and used to design new inhibitors of an important protein–protein interaction.

## 

Aurora A is one of three human aurora kinases, a family of serine/threonine kinases that play a central role in cell division.^1^ Aurora A and Aurora B are critical for mitotic cell division, in which Aurora C is also implicated^2^ alongside its role in meiosis.^3^ During mitosis, Aurora A associates with the centrosome and the spindle microtubules to control centrosome maturation and spindle assembly.^4^ It acts in part through direct phosphorylation of partners such as PLK1.^5^ Aurora A is ubiquitously expressed but its expression is strongly cell cycle dependent. Its expression peaks at the G2-M transition, when it is involved in the mitotic checkpoint.^6^ Aurora A is a 403-residue protein, composed of an N-terminal domain, a protein kinase domain, and a C-terminal domain. The N-terminal and C-terminal domains contain a KEN degradation motif and a destruction box (D-box) respectively, both of which control degradation.^7^ Aurora A is oncogenic and is overexpressed in tumors of the breast, colon, stomach, and ovaries.^8^ Inhibition of Aurora A leads to cell death in dividing cells, through a mechanism involving chromosome misalignment and stalling at the mitotic checkpoint.^9^,^10^ As a consequence, it has received a lot of attention as a potential drug target in cancer^7^ and numerous kinase inhibitors have been described.^11^–^13^ A number of these inhibitors are now in clinical trials.^11^ As well as the ATP-binding site, an additional allosteric binding site can also be targeted to modulate Aurora A function.^14^ During mitosis, Aurora A is localized to microtubules in the mitotic spindle through an interaction between the kinase domain and the protein TPX2.^15^ The N-terminal sequence of TPX2 binds to an allosteric pocket on Aurora A^16^ and stimulates kinase activity, leading to cell-cycle progression. Interruption of the Aurora A–TPX2 interaction reduces kinase activity, leading to mislocalization of Aurora A, mitotic defects, and cell cycle arrest.^17^

In previous work, some of us have described the development of small-molecule inhibitors targeting the TPX2 binding pocket of Aurora A.^18^ In particular, through a process of high-throughput screening of diverse chemical libraries^19^ and fragment deconstruction, the fragment 2-phenyl-4-carboxyquinoline (compound **1**, Fig. 1) was developed. Compound **1** shows a dose-dependent inhibition of TPX2 binding to Aurora A in a fluorescence anisotropy (FA) assay (*K*_i_ = 63 μM). A process of synthesis and investigation of structure–activity relationship (SAR) trends was embarked upon to improve the potency of the fragment, mainly through increasing the hydrophobicity of the phenyl group which occupies the pocket formed by residues L178, V182, V206 and L208 (Fig. 1). The most potent molecule, named AurkinA (*K*_i_ = 2.7 μM), was shown to inhibit the kinase activity of Aurora A *in vitro* and mislocalize Aurora A from mitotic spindle microtubules *in vivo*.^18^ AurkinA provides a blueprint for future design efforts that target the TPX2 binding pocket of Aurora A.

**Fig. 1:**
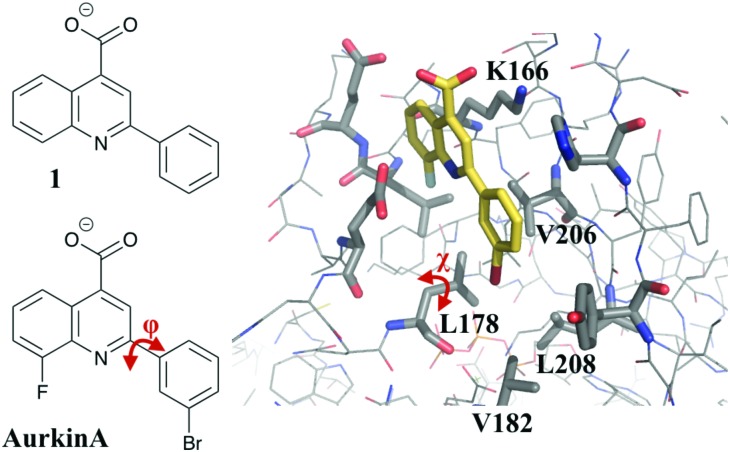
Crystal structure of AurkinA (compound **13**) bound to Aurora A kinase.^18^ Flexible torsional angles in the ligand (*φ*) and L178 on the protein (*χ*) are shown in red.

Free energy perturbation (FEP) theory for the computation of relative binding free energies is a promising companion for the fragment-based drug discovery (FBDD) techniques used above (see the ESI†). In FBDD, improvement of the binding affinity is the most important factor and multiple rounds of synthesis are often required to obtain the requisite potency gains.^20^ However, for fragments that bind with mM to high μM affinity it is often difficult to accurately measure binding constants in biological assays and hence pursue SAR. These technical problems should not affect the accuracy of FEP methods and hence efficiency gains in the FBDD process are expected if X-ray crystallography and computational predictions are used in tandem. A recent study of the binding of 90 small molecules to a range of targets revealed that FEP is able to predict relative binding affinities in FBDD studies with root mean square (RMS) errors of approximately 1.1 kcal mol^–1^.^21^

The MCPRO software^22^ is a widely used tool for quantitative predictions of relative binding free energies, which uses FEP theory in combination with Monte Carlo sampling (MC/FEP). Notable successes include the prediction of a number of extremely potent inhibitors of HIV reverse transcriptase^23^ and macrophage migration inhibitory factor.^24^ However, it has not been used before now to our knowledge in FBDD efforts. A potential hurdle to its uptake is the possibility of a range of dynamic binding modes that are available to small, flexible fragments. In the current study for example the flexibility of the phenyl-quinoline linker (Fig. 1) means that groups added to the phenyl ring may orient either toward or away from the hydrophobic floor of the binding pocket. Furthermore, as we shall show, the addition of the bulky groups to the ligand may induce re-orientation of the L178 side chain, in a manner reminiscent of the V111 side chain rotation in the T4 lysozyme model system, which has been shown to be problematic for standard FEP simulations.^25^

In what follows, we describe the application of MCPRO, in combination with the recently implemented replica exchange with solute tempering (REST) enhanced sampling method,^26^–^29^ to the study of the relative affinities of 14 small molecule inhibitors of Aurora A. The REST algorithm has been shown to substantially improve the consistency of MC/FEP results by improving conformational sampling, thereby reducing dependency on the choice of starting structure.^28^ The computed conformational ensembles have additionally been shown to be in excellent agreement with molecular dynamics simulations.^29^ Here, the REST method successfully incorporates torsional sampling of both the phenyl group and L178 side chain in a single simulation and recapitulates the experimental binding data in the majority of cases. In addition, we make two new experimentally-verified predictions, one of which yields a small molecule inhibitor that is equally potent as AurkinA. This study points to the potential for routine use of MC/FEP in prospective fragment-based lead optimization.

Optimization of the binding potency of small molecule analogs of compound **1** was initially pursued by investigating substituents at the *meta* and *para* positions of the phenyl ring (see the ESI†). The asymmetric substitutions pose a problem for traditional FEP simulations, since the simulation firstly needs to find the preferred binding pose (*e.g.*Fig. 2(a and b)), which is not necessarily known *a priori*, and secondly needs to account for the entropic penalty associated with the loss of symmetric binding modes.^30^ The REST algorithm implemented in MCPRO accounts for both of these factors by effectively enhancing the sampling of dihedral angle ‘flips’ between alternative binding modes.^28^

**Fig. 2:**
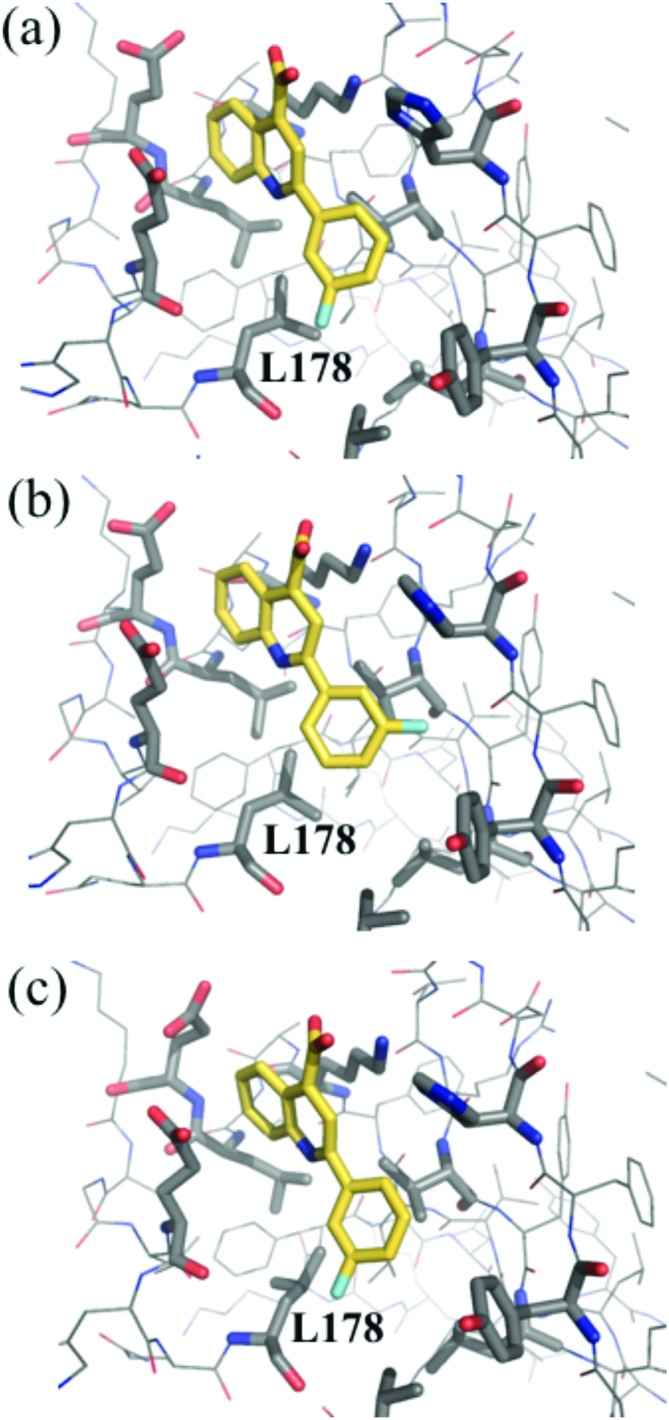
Examples of structures sampled during MC/FEP simulations of compound **2** bound to Aurora A. (a) *φ* = 180°, *χ* = 180°, (b) *φ* = 330°, *χ* = 180°, (c) *φ* = 180°, *χ* = 60°.

In addition, our crystallographic data are inconclusive concerning which of the two rotamers of L178 shown in Fig. 2(a and c) is preferred for a given substituent. Previous crystallographic studies of the T4 lysozyme hydrophobic cavity have shown that the size of the binding pocket is strongly influenced by the size of the bound ligand^31^ and computational estimates of binding affinity can be strongly dependent on the choice of starting structure.^25^,^32^ Here, initial estimates of the binding free energy of a Cl substituent at the *meta* position, relative to F, gave –0.27 kcal mol^–1^ starting from the structure shown in Fig. 2(a) and –0.78 kcal mol^–1^ starting from the structure in Fig. 2(c). We have therefore added the residue L178 to the REST enhanced sampling region and allowed flips in the angle *χ* during our simulations (Fig. 1). The computed binding free energy of Cl, relative to F, is then independent of the choice of starting structure (–0.73 and –0.80 kcal mol^–1^ respectively).

Table 1 shows the comparisons between computation (including both the ligand and residue L178 in the REST region) and experimental FA assays.^18^ In general, it can be seen that adding halogens at the position X is predicted to be favorable. In particular, with the enhanced sampling of L178, the prediction Br > F > H is in line with experimental results. X = Cl is actually predicted to be more potent than X = Br, but compound **4** has not been synthesized. The additional substitution of Z = F is also found to enhance binding relative to Z = H.

**Table 1:** Comparisons between computed relative free energies of binding (ΔΔ*G*) and experiment^18^

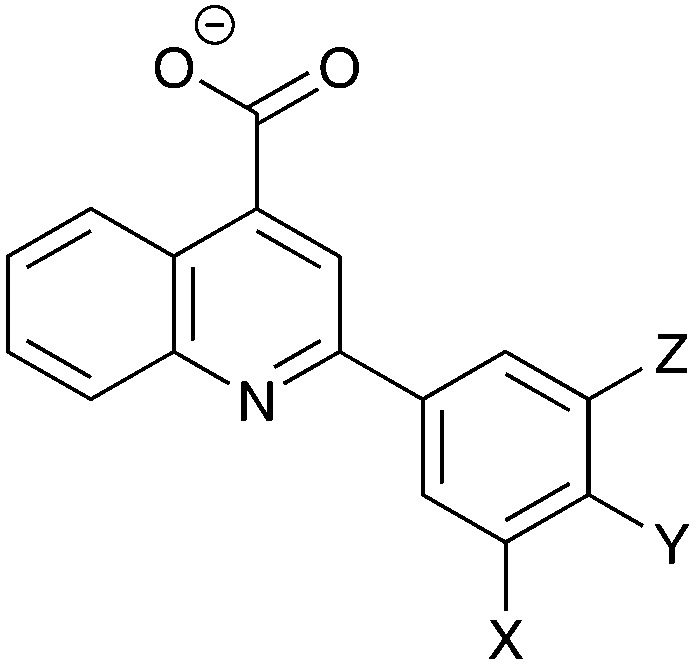						
	X	Y	Z	ΔΔ*G*^*a*^	IC_50_^*b*^	*K*_i_ ^*b*^
**1**	H	H	H	1.05	289	62.5
**2**	F	H	H	0.00	75.9	16.5
**3**	F	H	F	–0.94	36.0	7.8
**4**	Cl	H	H	–0.73	ND	ND
**5**	Cl	H	F	–0.89	20.5	4.4
**6**	Br	H	H	–0.49	25.6	5.5
**7**	CF_3_	H	H	0.11	26.5	5.7
**8**	CH_3_	H	H	1.12	ND	ND
**9**	F	CH_3_	H	–0.49	42^*c*^	8.7^*c*^
**10**	Br	CH_3_	H	–0.90	11.1^*c*^	2.3^*c*^

^*a*^kcal mol^–1^.

^*b*^μM.

^*c*^This work (see the ESI).

To demonstrate the conformational sampling facilitated by the REST method, Fig. 3 shows the distribution of dihedral angles in simulations of **2** and **5** bound to Aurora A. Compound **2** samples all of the conformations shown in Fig. 2 with a preference for *φ* = 330° and *χ* = 180° (Fig. 2(b)). In contrast, binding of **5** with the bulkier Cl in the *meta* position leads to a reorientation of the L178 side chain (*χ* = 60°). There is a slight preference for Cl to be oriented toward the hydrophobic floor of the binding pocket (*χ* = 180°) but both conformations of the phenyl group are sampled. In order to check the orientation of the small molecules in the binding pocket, we have collected X-ray crystal structures of **2** and **5** (see the ESI†). In contrast to the MC simulations, both crystal structures clearly show that *φ* is close to 180°. The discrepancy is possibly due to force field inaccuracy, crystallization conditions, or a small population of alternative conformations may not be visible in the X-ray electron density. It is more difficult to assign the orientation of the L178 side chain, and so the inter-conversion between the two populations observed in the MC simulations may be accurate.

**Fig. 3:**
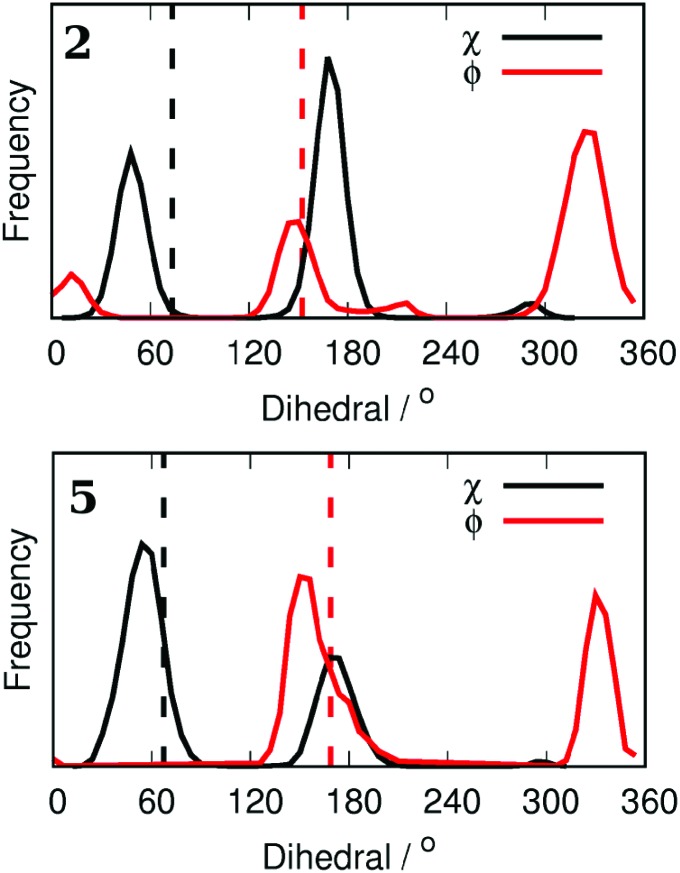
Dihedral angle distributions from Monte Carlo simulations for both the phenyl ring of compounds **2** and **5** (*φ*) and the side chain of L178 (*χ*). Assigned dihedral angles from X-ray crystal structures are displayed as vertical dashed lines.

Returning to the prediction of relative binding free energies, the substitution of bulky methyl and trifluoromethyl at the *meta* position was predicted to be less favorable than compound **1**, although **7** showed reasonable activity experimentally (Table 1). On the basis of the computational data **8** was not pursued further. Interestingly, however, there does appear to be space to accommodate a methyl substitution in the TPX2 pocket at the *para* position on the phenyl ring. In particular, compounds **9** and **10** show enhanced activity in FEP simulations relative to **2** and **6**, respectively. As a result of these predictions, compounds **9** and **10** were synthesized and assayed (see the ESI†), resulting in the most potent fragment reported here (compound **10**, *K*_i_ = 2.3 μM). As predicted, compound **9** also shows enhanced activity relative to **2**.

Encouraged by these results, we tested four more compounds with potential benefits (Fig. 4). Compound **11** introduces an electronegative N atom into the TPX2 binding pocket, but this is predicted to be unfavorable. This result is in qualitative agreement with the SAR presented in ref. 18 for a set of related molecules. Compound **12** was expected to orient the ligand for optimal binding in the Aurora A binding pocket. However, the relative binding free energy was predicted to be similar to **6** and so was not pursued further. As a further test of the FEP methodology, the relative binding free energy of AurkinA (compound **13**) was computed. In agreement with experiment, the F substituent on the quinoline was extremely favorable. A similar substitution on compound **10** may improve its potency still further. Finally, compound **14** showed good predicted activity but could not be synthesized so far.

**Fig. 4:**
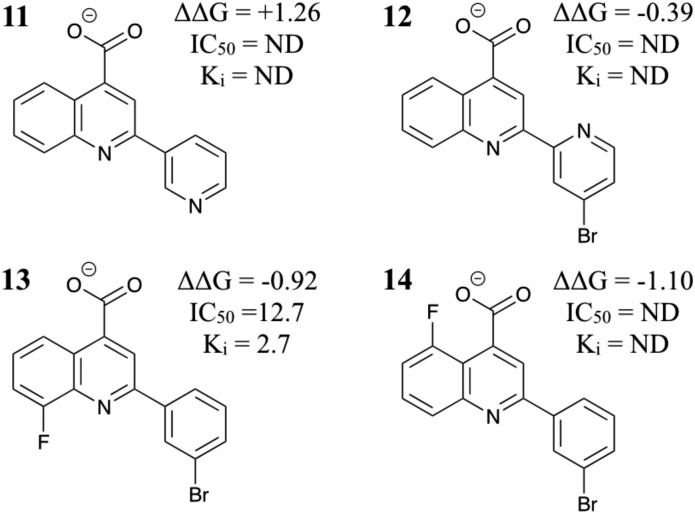
Additional MC/FEP relative free energies of binding (kcal mol^–1^) and experimental results where available.^18^

As a means of benchmarking computational methodologies, it is commonplace to compare computed ΔΔ*G* with experimental pIC_50_.^21^,^30^ Fig. 5 compares the computed and experimental results where available. The mean unsigned error is 0.24 kcal mol^–1^, the root-mean-square error is 0.32 kcal mol^–1^, and the Pearson correlation coefficient is 0.86. The largest errors are for compounds **3** and **7**, but even these are relatively small. Perhaps most encouragingly for the use of FEP as a computational pre-screening tool, the three most potent fragments (**5**, **10** and **13**) are in the top four computational predictions.

**Fig. 5:**
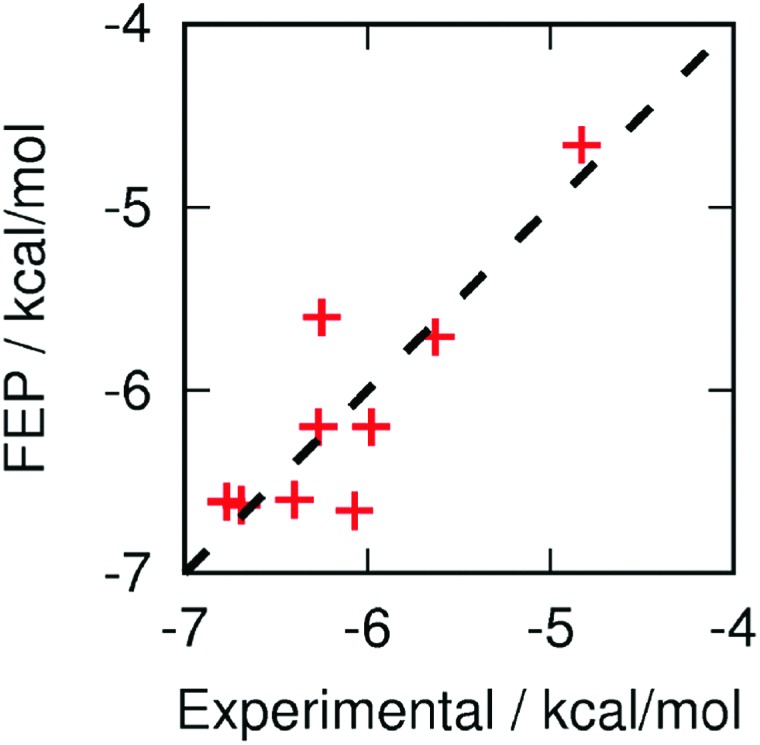
Correlation between computed and experimental (= RT ln(IC_50_)) binding free energies. Computational results are offset to give the same mean as the experimental distribution.

In summary, the replica exchange with solute tempering method for the enhanced sampling of both protein and small molecule degrees of freedom has been implemented in MCPRO and used for the first time in FBDD to rank inhibitors of the Aurora A–TPX2 protein–protein interaction. The utility of the method is demonstrated by the experimentally-verified prediction of two novel small-molecule inhibitors (compounds **9** and **10**), one of which is as potent as AurkinA.^18^ More generally, FEP shows promise as a pre-screening tool for use in prospective fragment-based drug design efforts, especially when combined with enhanced sampling of protein–ligand binding modes.

There are no conflicts to declare. This project was supported by a Marie Curie International Outgoing Fellowship within the seventh European Community Framework Programme, a Wellcome Trust Strategic Award 090340/Z/09/Z to ARV, DRS and MH, the Medical Research Council programme grants MC_UU_12022/1 and MC_UU_12022/8 and grant ML/L007266/1 to ARV, and a National Institutes of Health grant GM32136 to WLJ.

## Supplementary Material

Supplementary information

Supplementary information
