# Supplementary material for: Computationally-guided optimization of small-molecule inhibitors of the Aurora A kinase–TPX2 protein–protein interaction
Source: Chem Commun (Camb). 2017 Aug 2;53(67):9372–5. doi: 10.1039/c7cc05379g (PMC5591577; doi:10.1039/c7cc05379g)
Supplement: Supplementary file 1 [file CC-053-C7CC05379G-s001.pdf]

Electronic Supplementary Material (ESI) for ChemComm.
This journal is © The Royal Society of Chemistry 2017

Electronic supplementary information for:

Computationally-Guided Optimization of Small-Molecule Inhibitors of the Aurora A Kinase - TPX2 Protein-Protein Interaction

Daniel J. Cole,[1,2] Matej Janecek,[3,4] Jamie E. Stokes,[4] Maxim Rossmann,[5] John C. Faver,[1] Grahame J. McKenzie,[3] Ashok R. Venkitaraman,[3] Marko Hyvönen,[5] David R. Spring,[4] David J. Huggins,[3,4,6] William L. Jorgensen[1]

[1] Department of Chemistry, Yale University, New Haven, Connecticut 06520-8107, United States.

[2] School of Chemistry, Newcastle University, Newcastle upon Tyne NE1 7RU, United Kingdom.

[3] MRC Cancer Unit, University of Cambridge, Hills Road, Cambridge CB2 0XZ, United Kingdom.

[4] Department of Chemistry, University of Cambridge, Lensfield Road, Cambridge CB2 1EW, United Kingdom. [5] Department of Biochemistry, University of Cambridge, 80 Tennis Court Road, Old Addenbrooke's Site, Cambridge CB2 1GA, United Kingdom. [6] Theory of Condensed Matter Group, Cavendish Laboratory, 19 JJ Thomson Avenue, Cambridge CB3 0HE, United Kingdom.

1. COMPUTATIONAL METHODS

Free energy perturbation (FEP) theory is a computational technique, which is used in conjunction with molecular dynamics or Monte Carlo sampling to determine protein-ligand free energies of binding [1-3]. In principle, FEP includes all components of the binding free energy, including changes in ligand and receptor conformation, hydration penalties and changes in entropy. The predictive power of FEP is limited only by the accuracy of the underlying molecular mechanics force field and the finite sampling of conformational degrees of freedom. Both of these limitations are undergoing continuing improvement, and the current manuscript addresses the latter to some extent by enhancing the sampling of ligand and receptor degrees of freedom.

The rigor of FEP calculations does come with disadvantages. Compared to other computational tools that are more common in fragment-based drug design, such as fragment-based active site mapping, virtual screening of fragment libraries, and de novo fragment-based growing/linking (reviewed in Refs. [4,5]), the computational expense and required user expertise of FEP is higher. However, with the increasing use of graphical processing units (GPUs) to reduce simulation times and automated set-up of calculations to reduce user errors [6-8], we envisage FEP becoming even more widely used alongside traditional computational methods and experiment for fragment-based drug design [9].

In this communication, FEP simulations followed standard protocols [10-13]. Initial coordinates were constructed from the X-ray crystal structure of compound **2** bound to Aurora A using the MCPRO software [1]. ATP, counter-ions and crystal water molecules were removed from the structure. The model comprised the 113 residues closest to the ligand. Residues within a 10 Å radius sphere were allowed to move during the simulations and backbone moves were controlled by the concerted rotation algorithm [15]. The unbound ligand and complexes were solvated in a 25 Å water cap. The latter used the JAWS algorithm [16] with standard parameter settings to place water molecules optimally around the binding site. The protein energetics were described using the OPLS/AA force field, the ligands with OPLS/CM1A [17] and water with TIP4P. FEP calculations used 11 λ windows of simple overlap sampling [18]. Each window consisted of between 10 and 30 million (M) configurations of equilibration and 40M/50M configurations of averaging for the bound/unbound simulations.

At each λ window, four replicas were run in parallel with replica exchange with solute tempering (REST) enhanced sampling applied to the ligand and, as described in the main text, the protein residue

L178 [12,13]. The REST method is implemented in MCPRO as described in Ref. [12] with the following modifications:

- Intramolecular angle bending force field terms are no longer re-scaled. That is, the REST scaling factor is applied only to dihedral and non-bonded terms. This modification significantly increases replica exchange rates without adversely affecting conformational sampling.
- Any protein residue may now be selected for enhanced sampling, as well as the ligand. That is, the dihedral rotation and non-bonded force field terms of the selected residue are now effectively re-scaled to reduce potential energy barriers to rotation in high temperature replicas of the system [12]. Since the number of atoms in the REST region is now higher, care must be taken to ensure adequate acceptance rates for replica exchange.

REST scaling factors were chosen to be exponentially distributed (25, 86, 160, 250 ^{o}C). With these replica temperatures, replica exchange was in the range 15-30% for all λ windows. Exchange attempts between pairs of neighboring replicas were attempted every 10 000 MC steps. The REST protocol was used alongside the 'flip' algorithm in which selected dihedral angles undergo attempted jumps that are much larger than typical MC moves [12,19]. The flip algorithm was applied to the side chain χ_2 dihedral angle in L178 and the angle between the phenyl ring and quinoline core of the ligand (χ and φ in Figure 1 of the main text). The jumps were of random size in the range 60° to 300°.

2. CHEMICAL SYNTHESIS OF AURORA A KINASE-TPX2 INHIBITOR ANALOGS

Synthesis of compounds **1-8** proceeded as described in Ref [20]. Synthesis of **9** and **10** is described below.

2-(3-fluoro-4-methylphenyl)quinoline-4-carboxylic acid (9)

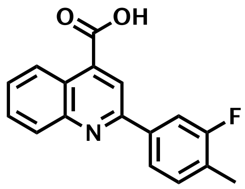

Isatin (250 mg, 1.70 mmol), 3-fluoro-4-methylacetophenone (310 mg, 2.04 mmol) and KOH (286 mg, 5.10 mmol) were suspended in EtOH (3.5 mL) and the resulting mixture heated to reflux overnight. The mixture was allowed to cool and the solvent was removed under reduced pressure. The residue was dissolved in H_2O and washed with an equal volume of Et_2O (x2). The aqueous fraction was acidified to pH 1 with 3N aqueous HCl. The precipitate was collected by filtration and recrystallized from MeOH to furnish the title compound **9** as an orange solid (53 mg, 0.19 mmol, 11%).

^{1}H NMR (400 MHz; d_6-DMSO) 8.63 (dd, *J* = 9.0, 1.0 Hz, 1H), 8.46 (s, 1H), 8.16 (dd, *J* = 9.0, 1.0 Hz, 1H), 8.08 - 8.03 (m, 2H), 7.88 – 7.83 (m, 1H), 7.73 – 7.69 (m, 1H), 7.47 (t, *J* = 8.0 Hz, 1H), 2.32 (s, 1H); **^{13}C NMR** (100 MHz; d_6-DMSO) 167.5, 161.2 (*J* = 241.0 Hz), 154.4 (*J* = 2.5 Hz), 148.1, 137.9, 137.7 (*J* = 7.5 Hz), 132.2 (*J* = 5.0 Hz), 130.4, 129.6, 127.9, 126.6 (*J* = 17.0 Hz), 125.4, 123.5, 123.0 (*J* = 3.0 Hz), 118.9, 113.3 (*J* = 24.0 Hz), 14.2 (*J* = 3.0 Hz); **IR** ν_{max} (neat/cm^{-1}) 3500-2360 (OH, broad), 1727 (C=O, sharp); **HRMS** (ESI+) *m/z* found $[M+H]^+$ 282.0920, $C_{17}H_{13}O_2NF$ required 282.0925.

2-(3-bromo-4-methylphenyl)quinoline-4-carboxylic acid (10)

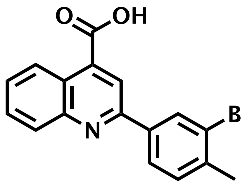

Isatin (500 mg, 3.40 mmol), 3-bromo-4-methylacetophenone (870 mg, 4.08 mmol) and KOH (572 mg, 10.2 mmol) were suspended in EtOH (7.0 mL) and the resulting mixture heated to reflux overnight. The mixture was allowed to cool and the solvent was removed under reduced pressure. The residue was dissolved in H_2O and washed with an equal volume of Et_2O (x2). The aqueous fraction was acidified to pH 1 with 3N aqueous HCl. The precipitate was collected by filtration and recrystallized from MeOH to furnish the title compound **10** as an orange solid (149 mg, 0.44 mmol, 13%).

^{1}H NMR (400 MHz; *d*$_6$-DMSO) 14.0 (br s, 1H), 8.63 (dd, *J* = 8.5, 0.5 Hz, 1H), 8.52 (d, *J* = 1.5 Hz, 1H), 8.46 (s, 1H), 8.21 (dd, *J* = 8.0, 2.0 Hz, 1H), 8.17 (d, *J* = 8.0 Hz, 1H), 7.87 – 7.83 (m, 1H), 7.73 – 7.69 (m, 1H), 7.54 (d, *J* = 8.0 Hz, 1H), 2.43 (s, 1H); **^{13}C NMR** (100 MHz; *d*$_6$-DMSO) 167.6, 154.1, 148.3, 139.3, 137.9, 137.6, 131.7, 130.5, 130.4, 129.9, 128.0, 126.4, 125.4, 125.1, 123.6, 119.0, 22.4; ν_{max} (neat/cm^{-1}) 3200-2200 (OH, broad), 1692 (C=O, sharp); **HRMS** (ESI+) *m/z* found $[M+H]^+$ 342.0120, $C_{17}H_{13}O_2NBr$ required 342.0130.

3. FLUORESCENCE POLARIZATION ASSAY

The fluorescence polarization assay as well as the procedure to obtain the protein (Hisx6-Aurora $A_{123\text{-}403}$), were described in detail and validated in Ref [20]. To summarize, the fluorescence polarisation (FP) measurements were carried out in a 384-well, low-volume, black, flat bottom polystyrene NBS microplate (Corning 3820) using a PHERAstar Plus plate reader (BMGLabtech). The polarization values are reported in arbitrary millipolarization units (mP) and were measured at an excitation wavelength of 540 nm and an emission wavelength of 590 nm. The optimized assay used His-Aurora $A_{123\text{-}403}$ (300 nM), TAMRA-TPX$2_{1\text{-}43}$ (12 nM) in the assay buffer (PBS, 0.3 mM DTT). The Z-factor for the TAMRA-TPX$2_{1\text{-}43}$ assay was calculated to be 0.66-0.71. The total volume per well

was 30 μL. Compounds were subjected to assays at decreasing concentrations by serial dilutions and two (HTS) to four replicates were measured. The binding curves were analyzed using GraphPad Prism 6.0 software. Non-linear regression curve fit with variable slope was used to yield IC_{50} (competitive binding) and K_d values (direct binding, no receptor depletion). Lower limit constraint was imposed on the analysis to match the mP of the TAMRA-TPX2 peptide only control. K_i values were calculated from IC_{50} using model equations as defined by Nikolovska-Coleska *et al*. [21]. The displacement of TAMRA-$TPX2_{1-43}$ peptide from Aurora A protein upon the titration of the compounds **6** (described previously, [20]), **9** and **10** is shown in Supplementary Figure 1.

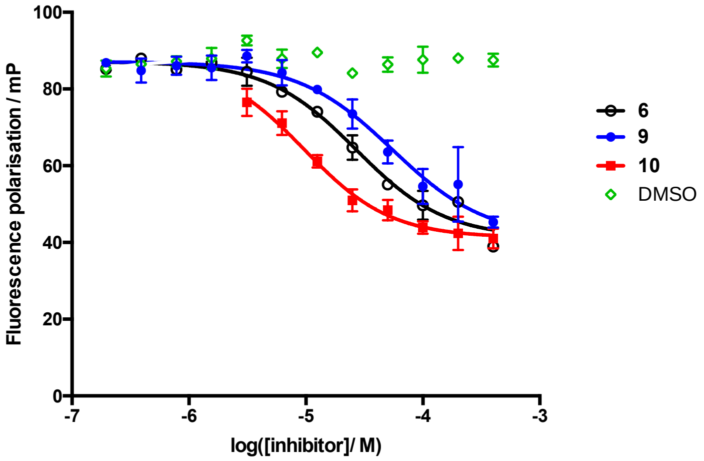

Supplementary Figure 1: The fluorescence polarization assay of compounds **9** and **10** (**6** included for comparison). The inhibition curves were fitted to the data using GraphPad Prism 6.0 and IC_{50} values were determined. The corresponding K_i values were subsequently calculated [21].

4. CRYSTALLOGRAPHIC DATA

Production of Aurora A for crystallography, crystallization ligand soaking and structure determination was performed as described previously [20]. Structure determination statistics are shown in Supplementary Table 1. The coordinates and associated structure factors have been submitted to the Protein Data Bank under accession numbers 5OBJ (Aurora A in complex with compound **2**) and 5OBR (Aurora A in complex with compound **5**).

Supplementary Table 1. Crystallographic data collection, processing and refinement statistics.

Protein	Aurora A kinase	Aurora A kinase
Ligand	**2 + ATP**	**5 + JNJ-7706621**
PDB code	5OBJ	5OBR
**Data collection**		
Synchrotron and beamline	DLS, i04-1	Soleil, Proxima-1
Wavelength (Å)	0.9200	0.9184
Temperature (K)	100.0	100.0
**Data processing**		
Resolution (Å)	72.24 - 2.90 (3.00- 2.90)	41.85-2.62 (2.77-2.62)
Space group	$P6_1$ 2 2	$P6_1$ 2 2
Unit cell: a,b,c (Å)	83.41, 83.41, 172.09	83.70, 83.70, 169.70
α,β,γ (deg)	90.0, 90.0, 120.0	90.0, 90.0, 120.0
R_{merge}	0.017 (0.188)	0.046 (0.606)
R_{meas}	0.066 (0.731)	0.052 (0.674)
No. of unique observations	62971 (4921)	58358 (9164)
Total number unique	8428 (604)	11215 (1735)
Mean((I)/σ(I))	24.7 (3.5)	26.20 (2.38)
$CC_{1/2}$	1 .0 (0.90)	1.0 (0.85)
Completeness (%)	99.8 (100)	99.8 (99.1)
Multiplicity	7.5 (8.1)	5.2 (5.3)
**Refinement**		
Resolution (Å)	55.32-2.90 (3.32-2.90)	41.85-2.62 (2.88-2.62)
R_{work}	0.214 (0.268)	0.223 (0.292)
R_{free}	0.246 (0.296)	0.292 (0.365)
No. of non-H atoms	2186	2130
Protein atoms	2125	2127
Ligand atoms	58	48
Waters	3	15
RMSD bonds (Å)	0.01	0.01
RMSD angles (deg)	1.19	1.17
Ramachandran favored (%)	93.7	94.8
Ramachandran allowed (%)	5.9	3.6
Ramachandran outliers (%)	0.4	1.6
Molprobity clashscore	6.49	5.77
Average B-factor (Å^2)		
of all atoms	74.2	68.7
of macromolecules	74.4	68.8
of ligands	66.3	56.8

REFERENCES

[1] Jorgensen, W. L. *Acc. Chem. Res.* **2009**, *42*, 724-733.

[2] Chodera, J. D.; Mobley, D. L.; Shirts, M. R.; Dixon, R. W.; Branson, K.; Pande, V. S. *Curr. Opin. Struct. Biol.* **2011**, *21*, 150-160.

[3] Hansen, N.; van Gunsteren, W. F. *J. Chem. Theory Comput.* **2014**, *10*, 2632-2647.

[4] Sheng, C.; Dong, G.; Wang, C. *Methods in Pharmacology and Toxicology* **2016**, 189-215.

[5] Kumar, A.; Voet, A.; Zhang, K. Y. J. *Curr. Med. Chem.* **2012**, *19*, 5128-5147.

[6] Wang, L.; Wu, Y.; Deng, Y.; Kim, B.; Pierce, L.; Krilov, G.; Lupyan, D.; Robinson, S.; Dahlgren, M.; Greenwood, J.; Romero, D. L.; Masse, C.; Knight, J. L.; Steinbrecher, T.; Beuming, T.; Damm, W.; Harder, E.; Sherman, W.; Brewer, M.; Wester, R.; Murcko, M.; Frye, L.; Farid, R.; Lin, T.; Mobley, D. L.; Jorgensen, W. L.; Berne, B. J.; Friesner, R. A.; Abel, R. *J. Am. Chem. Soc.* **2015**, *137*, 2695−2703.

[7] Liu, S.; Wu, Y.; Lin, T.; Abel, R.; Redmann, J. P.; Summa, C. M.; Jaber, V. R.; Lim, N. M.; Mobley, D. L. *J. Comput. Aided Mol. Des.* **2013**, *27*, 755–770.

[8] Loeffler, H. H.; Michel, J.; Woods, C. *J. Chem. Inf. Model.* **2015**, *55*, 2485-2490.

[9] Schmidt, T. C.; Eriksson, P.-O.; Gustafsson, D.; Cosgrove, D.; Frølund, B.; Boström, J. *J. Chem. Inf. Model.* **2017**, *57*, 1703-1714.

[10] Bollini, M.; Domaoal, R. A.; Thakur, V. V.; Gallardo-Macias, R.; Spasov, K. A.; Anderson, K. S.; Jorgensen, W. L. *J. Med. Chem.* **2011**, *54*, 8582-8591.

[11] Dziedzic, P.; Cisneros, J. A.; Robertson, M. J.; Hare, A. A.; Danford, N. E.; Baxter, R. H. G.; Jorgensen, W. L. *J. Am. Chem. Soc.* **2015**, *137*, 2996-3003.

[12] Cole, D. J.; Tirado-Rives, J.; Jorgensen, W. L. *J. Chem. Theory Comput.* **2014**, *10*, 565-571.

[13] Cole, D. J.; Tirado-Rives, J.; Jorgensen, W. L. *Biochim. Biophys. Acta* **2015**, *1850*, 966-971.

[14] Jorgensen, W. L. *Acc. Chem. Res.* **2009**, *42*, 724-733.

[15] Ulmschneider, J. P.; Jorgensen, W. L. *J. Chem. Phys.* **2003**, *118*, 4261-4271.

[16] Luccarelli, J.; Michel, J.; Tirado-Rives, J.; Jorgensen, W. L. *J. Chem. Theory Comput.* **2010**, *6*, 3850-3856.

[17] Udier-Blagovic, M.; Morales De Tirado, P.; Pearlman, S. A.; Jorgensen, W. L. *J. Comput. Chem.* **2004**, *25*, 1322-1332.

[18] Jorgensen, W. L.; Thomas, L. L. *J. Chem. Theory Comput.* **2008**, *4*, 869-876.

[19] Thomas, L. L.; Christakis, T. J.; Jorgensen, W. L. *J. Phys. Chem. B* **2006**, *110*, 21198-21204.

[20] Janeček, M.; Rossmann, M; Sharma, P; Emery A.; Huggins, D. J.; Stockwell, S. R.; Stokes, J. E.; Tan, Y. S.; Almeida, E. G.; Hardwick, B.; Narvaez, A. J.; Hyvönen, M.; Spring, D. R.; McKenzie, G. J.; Venkitaraman, A. R. *Sci. Rep.* **2016**, *6*, 28528

[21] Nikolovska-Coleska, Z.; Wang, R.; Fang, X.; Pan, H.; Tomita, Y.; Li, P.; Roller, P. P.; Krajewski, K.; Saito, N. G.; Stuckey, J. A. *Anal. Biochem.* **2004**, *332*, 261–273.
